# Exploring associations of greenery, air pollution and walkability with cardiometabolic health in people at midlife and beyond

**DOI:** 10.1111/ggi.14743

**Published:** 2023-12-19

**Authors:** Alison Carver, Richard Beare, Luke D Knibbs, Suzanne Mavoa, Kaya Grocott, Amanda J Wheeler, Velandai Srikanth, Nadine E Andrew

**Affiliations:** ^1^ National Centre for Healthy Ageing Melbourne Victoria Australia; ^2^ Peninsula Clinical School, Central Clinical school, Faculty of Medicine Monash University Melbourne Victoria Australia; ^3^ Peninsula Health Melbourne Victoria Australia; ^4^ Developmental Imaging Murdoch Children's Research Institute Melbourne Victoria Australia; ^5^ School of Public Health The University of Sydney Sydney New South Wales Australia; ^6^ Public Health Research Analytics and Methods for Evidence, Public Health Unit Sydney Local Health District Camperdown New South Wales Australia; ^7^ Environmental Protection Authority Melbourne Victoria Australia; ^8^ University of Melbourne Melbourne Victoria Australia; ^9^ CSIRO Aspendale Victoria Australia

**Keywords:** air pollution, cardiometabolic disease, greenery, older adults, walkability

## Abstract

**Aim:**

To examine associations of neighborhood greenery, air pollution and walkability with cardiometabolic disease in adults aged ≥45 years in the Frankston–Mornington Peninsula region, Victoria, Australia.

**Methods:**

A cross‐sectional, ecological study design was used. We assessed mean annual neighborhood greenery using the Normalized Difference Vegetation Index; air pollution (fine particulate matter of diameter ≤2.5 μm [PM_2.5_] and NO_2_) using land‐use regression models; and walkability using Walk Score (possible values 0–100). Medically diagnosed diabetes (~95% type‐2), heart disease and stroke were self‐reported in the Australian Census (2021). Multivariable regression was used to model associations between environmental exposures and area‐level (neighborhood) cardiometabolic disease prevalence (age group ≥45 years), with socioeconomic status, age and sex as covariates. Air pollution was examined as a mediator of associations between greenery and disease prevalence.

**Results:**

Our sample comprised 699 neighborhoods with the following mean (SD) values: Normalized Difference Vegetation Index 0.47 (0.09), PM_2.5_, 8.5 (0.6) μg/m^3^ and NO_2_, 5.2 (1.6) ppb. Disease prevalences were: heart disease, mean 8.9% (4.5%); diabetes, mean 10.3% (4.7%); and stroke, median 1.2% (range 0–10.9%). Greenery was negatively associated with diabetes (*β* = −5.85, 95% CI −9.53, −2.17) and stroke prevalence (*β* = −1.26, 95% CI −2.11, −0.42). PM_2.5_ and NO_2_ were positively associated with diabetes (*β* = 1.59, 95% CI 1.00, 2.18; *β* = 0.42, 95% CI 0.22, 0.62) and stroke prevalence (*β* = 0.15, 95% CI 0.01, 0.29; *β* = 0.06, 95% CI 0.01, 0.10). The association between greenery and diabetes was partially mediated by PM_2.5_ (mediated effect −5.38, 95% CI −7.84, −3.03).

**Conclusions:**

Greenery and air pollutants were associated with lower and higher prevalence, respectively, of self‐reported diabetes and, to a lesser extent, stroke. These ecological findings require further exploration with stronger, longitudinal study designs to inform public health policy and directions. **Geriatr Gerontol Int 2024; 24: 208–214**.

## Graphical abstract

We examined associations of neighborhood greenery, air pollution and walkability with cardiometabolic disease in adults ≥45 years in the Frankston–Mornington Peninsula region, Victoria, Australia. Greenery and air pollutants were associated with lower and higher prevalence, respectively, of self‐reported diabetes and, to a lesser extent, stroke. The association between greenery and diabetes was partially mediated by PM_2.5_.

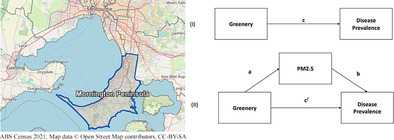

## Introduction

Cardiometabolic disease, including heart disease, stroke and type 2 diabetes, makes the greatest contribution to the global burden of disease.^1^ In 2019, 41 million people died from non‐communicable diseases, worldwide.^2^ Cardiovascular disease was the cause of 17.9 million of these deaths, while a further 2 million deaths were caused by diabetes.^2^ Although interventions that aim to lower modifiable risk factors for cardiometabolic disease are traditionally applied at the individual level, there is growing interest in the associations of local neighborhoods with these risk factors.^3^ Socioecological systems theory posits that there are multiple layers of influence centered on the individual related to their health.^4^, ^5^ There is increased awareness that built environment characteristics, such as clean air, the presence of trees and parks, and pedestrian/cycling infrastructure that promotes incidental physical activity, are important for the health of those who live, work and play there.^6^ As adults grow older and work less, they might spend more time in neighborhood settings that potentially influence their health.

A growing body of research suggests that air pollution might be adversely associated with cardiometabolic disease.^7^, ^8^, ^9^ For example, exposure to nitrogen dioxide (NO_2_), a key component of traffic emissions,^10^ has been associated with type 2 diabetes.^11^ In addition, exposure to fine particulate matter of diameter ≤2.5 μm (PM_2.5_), resulting from fossil fuel combustion, is considered a major contributor to global disease burden and reduced life expectancy due to cardiometabolic disease.^12^ Almost one‐third of all anthropogenic PM_2.5_ exposures come from vehicle emissions^10^ in urban areas of Australia, where car travel is the dominant transport mode.^13^ However, there is a scarcity of studies carried out in Australia on associations of air pollution and cardiometabolic disease.^7^, ^8^, ^9^ Even though there are generally lower concentrations of air pollutants in Australia, compared with low‐ to middle‐income countries, there is no safe threshold for pollutants.^14^ Therefore, examination of varying concentrations, even at lower levels, and associations with health‐related outcomes is warranted. Potentially, air pollution might be offset, at least in part, by greenery.^6^ Views of, and access to, greenery in urban areas are associated with lower levels of stress.^15^, ^16^ and higher levels of physical activity,^16^ which might protect against cardiometabolic disease. Public parks, in particular, provide settings for physical activity and interaction with nature.^17^

Another neighborhood characteristic that might promote residents' cardiometabolic health is walkability, which considers street connectivity, population density, and pedestrian access to shops and services.^18^ Walkable neighborhoods facilitate walking for transport to local destinations.^18^ A systematic review of longitudinal studies of the built environment and associations with cardiometabolic disease found that adults residing in more walkable neighborhoods were less likely to develop type 2 diabetes or hypertension.^19^ Another systematic review reported inverse associations between neighborhood walkability and risk/prevalence of type 2 diabetes, and between greenery and diabetes.^20^ Although parks and walkable neighborhoods show potential to promote cardiometabolic health, few studies have examined these environmental exposures in combination with air pollution.^21^ Further investigation is required, as more walkable neighborhoods might include more complex street networks with higher traffic volumes.^21^ Hence, it is vital to construct multi‐exposure models with health‐related outcomes to examine potential associations between co‐existing exposures.

To address these knowledge gaps, we aimed to explore multiple neighborhood environmental exposures in relation to the prevalence of self‐reported cardiometabolic disease among mid‐to‐older aged adults (aged ≥45 years) in the Frankston–Mornington Peninsula region, Australia (described under “Setting and sample”). Our specific questions were:

1. Are air pollutants positively associated with the prevalence of type 2 diabetes, heart disease and stroke?
2. Is greenery negatively associated with the prevalence of type 2 diabetes, heart disease and stroke?
3. Is greater walkability negatively associated with the prevalence of type 2 diabetes, heart disease and stroke?

We also explored the interaction between walkability and air pollution in relation to cardiometabolic disease prevalence, and air pollution as a potential mediator of associations between greenery and cardiometabolic disease prevalence.

## Methods

### Setting and sample

We carried out this analysis utilizing the health and environmental data infrastructure of the National Centre for Healthy Aging, an initiative funded by the Commonwealth Government of Australia.^22^ The study setting is the Frankston–Mornington Peninsula region (identified as Mornington Peninsula Statistical Area 4 [SA4] by the Australian Statistical Geography Standard),^23^ which contains a mixture of urban and regional locations.^24^ This area is shown in Appendix I, Figure A1. The total area is 853.8 km^2^, with most of this considered by the Australian Bureau of Statistics to be part of Melbourne's Significant Urban Area, comprising groups of urban centers (with population ≥10 000). However (as shown in Appendix I, Figure A2), much of the lower part of the Mornington Peninsula is considered to be regional.

The total population at the most recent 5‐yearly Census of Population and Housing (carried out in 2021) was 308 108, with 150 866 residents aged ≥45 years.^25^ We limited our age group of interest to ≥45 years, as cardiometabolic disease is more prevalent at midlife or older age.^1^ Our unit of analyses was the neighborhood; that is, we examined disease prevalence by neighborhood. Neighborhoods were operationalized at Statistical Area 1 (SA1) level, the smallest reporting unit of the Australian Census, representing approximately 200 households.^23^

### Outcome measures: Cardiometabolic disease prevalence in neighborhood

The 2021 Census^25^ was the first to ask respondents to report whether (yes/no) they had specific long‐term health conditions, diagnosed by a health professional. The following cardiometabolic disorders were included: “diabetes (non‐gestational),” “heart disease” and “stroke.” The number of people aged ≥45 years in each SA1 who reported having each disorder/disease was divided by the population in that age group, to give the disease prevalence, by SA1. Although the Census did not distinguish between type 1 and type 2 diabetes, almost all reported cases were assumed to be type 2, as it accounts for >96% of all diabetes cases globally.^26^

### Exposure measures

#### Greenery

Overall greenery (including tree canopy, grass) was assessed using the Normalized Difference Vegetation Index and satellite imagery (NASA Landsat 8, red and near‐infrared band). We included in our analysis mean annual Normalized Difference Vegetation Index values for each SA1 in 2019. These publicly available Normalized Difference Vegetation Index values were computed by Ramsay & Mavoa^27^ using Landsat sensor data, adjusted for variation in solar and atmospheric characteristics.

#### Air pollution

Exposures to annual average PM_2.5_ and NO_2_ were estimated for each SA1 using two validated satellite‐based land‐use regression models, developed for the Australian continent and described in detail elsewhere.^28^, ^29^ These model predictions were used to estimate exposures in 2019 at mesh block centroids (mesh block is the smallest area defined by ASGS), which aggregate to form whole SA1s, and we used the mesh blocks to average over each SA1 in our study area.^28^, ^29^

#### Walkability

The walkability of each SA1 was measured using Walk Score, a free online tool that computes a score (0–100) indicative of pedestrian access to local destinations (e.g. shops) from a chosen address.^30^ Walk Score assesses walking routes to destinations, with raw scores being assigned according to proximity (within 400 m, then up to 2.4 km), and accounts for population density and intersection density (a measure of street connectivity and route choice).^30^ Walk Score assigns higher scores to areas with better pedestrian access to local amenities, and has been validated internationally.^31^, ^32^ Walk Scores were generated for the address listed in the Geocoded National Address File^33^ closest to the centroid of each SA1, using the Walk Score Application Programming Interface with the R programming language.^34^

#### Covariates

To control for the socioeconomic status of the SA1, deciles of the Index of Relative Socioeconomic Disadvantage and Advantage were included.^35^ The Index of Relative Socioeconomic Disadvantage and Advantage is derived from Census data based on area‐level attributes of disadvantage (e.g. % unemployed) and advantage (e.g. % of employed classified as “Professionals”).^35^ Also, given that some disease risk factors increase with age and might vary by sex,^1^ our analyses were adjusted for the percentage of people aged ≥65 years and women.

### Statistical analysis

All statistical modelling (with significance set at *P* < 0.05) was carried out using IBM SPSS v26.0 (IBM Corporation, Armonk, NY, USA). Stroke prevalence was not normally distributed and underwent transformation (square root) before inclusion in analyses. Multicollinearity among exposure variables was assessed by examining variance inflation factors, and was considered to be present if variance inflation factors were >5.^36^ Initially, associations between each exposure and disease prevalence were examined in single exposure models using univariable linear regression. Subsequent multivariable regression analyses assessed cross‐sectional associations between environmental exposures and disease prevalence, controlling for the covariates described above. Further analyses were run for PM_2.5_ controlling for covariates and NO_2_, and vice versa. Potential interactions between walkability and air pollutants, proposed by a recent study,^21^ were examined in relation to disease prevalence, with effects scaled to one unit of each exposure.

MacKinnon's product of coefficients test^37^ was used to examine PM_2.5_ and NO_2_ as potential mediators of associations between greenery and disease prevalence. The standard methods^37^ are described in Appendix II, with pathways shown in Appendix II, Figure A3.

Ethical approval to carry out this study was not required, because the data had no individual identifiers, and were publicly available in the database of the 2021 Census of Population and Housing, in Australia.^25^

## Results

Our sample comprised 699 neighborhoods whose characteristics are described in Table 1.

**Table 1:** Characteristics of neighborhoods (Statistical Area 1)^†^

Characteristics	Neighborhoods (SA1s^†^; *n* = 699)	
Mean (SD) values for neighborhoods, unless otherwise stated		
% Female among those aged ≥45 years	52.8% (5.1%)	
Median area, km^2^ (range)	0.24 (0.05–60.91)	
Population density (people/km^2^)	1763 (1064)	
Median area‐level SES, IRSAD decile (range)	5 (1–10)	
Environmental exposures		
Greenery (NDVI; 0–1; unitless)	0.47 (0.09)	
PM_2.5_ (μg/m^3^)	8.5 (0.6)	
NO_2_ (ppb)	5.2 (1.6)	
Walk Score (0–100)	30.8 (21.5)	
Health conditions among those aged ≥45 years	No. people in neighborhood with health condition	Prevalence in neighborhood (%)
Diabetes	18.7 (11.9)	8.8% (4.5%)
Heart disease	20.0 (15.3)	8.9% (4.2%)
Stroke	Median 3, range 0–52	Median 1.2%, range 0–10.9%

Abbreviations: IRSAD, index of relative socioeconomic advantage and disadvantage; NDVI, Normalized Difference Vegetation Index; NO_2_, nitrogen dioxide; PM_2.5_, particulate matter of diameter ≤2.5 μm; ppb, parts per billion; SA1, Statistical Area 1; SES, socioeconomic status.

^†^ Analyses were conducted at SA1 level; however, for background information, the mean population for each SA1 was 440 (SD 152) people, with 215 (SD 90) people aged ≥45 years and 98 (SD 70) people ≥65 years.

Associations of environmental exposures with neighborhood prevalence of diabetes, heart disease and stroke among adults aged ≥45 years are presented below (Table 2). Significant associations were found between each of PM_2.5_, NO_2_ and greenery, and prevalence of diabetes. Higher levels of PM_2.5_ and NO_2_ were associated with higher prevalence of diabetes, whereas higher levels of greenery were associated with a lower prevalence of diabetes. Significant associations in the same corresponding directions were observed between these exposure variables and the transformed variable for stroke prevalence. Greenery was inversely associated with prevalence of heart disease in the univariable model. However, this association was no longer statistically significant after controlling for covariates (Table 2).

**Table 2:** Associations of environmental exposures with prevalence of cardiometabolic disease among adults aged ≥45 years, by Statistical Area 1

Exposure	Diabetes	Heart Disease	Stroke^†^
PM_2.5_ (μg/m^3^)	*β* [95% CI]	*β* [95% CI]	*β* [95% CI]
Unadjusted	***β* = 2.54 [2.03, 3.05]** ***	*β* = −0.18 [−0.69, 0.32]	*β* = 0.35 [−0.07, 0.14]
Adjusted for covariates^‡^	***β* = 1.59 [1.00, 2.18]** ***	*β* = 0.44 [−0.13, 1.00]	***β* = 0.15 [0.01, 0.29]** *
Adjusted for covariates and NO_2_ ^‡^	***β* = 1.41 [0.58, 2.25]** ***	*β* = 0.56 [−0.24, 1.36]	*β* = 0.07 [−0.13, 0.26]
NO_2_ (ppb)			
Unadjusted	***β* = 1.08 [0.89, 1.26]** ***	*β* = 0.08 [−0.11, 0.27]	***β* = 0.06 [0.02, 0.10]** **
Adjusted for covariates^‡^	***β* = 0.42 [0.22, 0.62]** ***	*β* = 0.08 [−0.12, 0.26]	***β* = 0.06 [0.01, 0.10]** *
Adjusted for covariates and PM_2.5_ ^‡^	*β* = 0.09 [−0.20, 0.37]	*β* = −0.60 [−0.33, 0.21]	*β* = 0.04 [−0.03, 0.10]
Greenery (mean NDVI)			
Unadjusted	***β* =** −**19.99 [−23.59, −16.40]** ***	***β* = −7.98 [−11.59, −4.36]** ***	***β* = −2.19 [−2.96, −1.43]** ***
Adjusted for covariates (excluding population density)^‡^ ^,^ ^§^	***β* = −5.85 [−9.53, −2.17]** **	*β* = **−**2.23 [−5.73, 1.26]	***β* = −1.26 [−2.11, −0.42]** **
Walkability (walk score)			
Unadjusted	***β* = 0.07 [0.06, 0.09]** ***	***β* = 0.04 [0.02, 0.05]** ***	***β* = 0.007 [0.004, 0.010]** ***
Adjusted for covariates^‡^	*β* =0.001 [−0.015, 0.016]	*β* = 0.00 [−0.015, 0.014]	*β* = 0.001 [−0.002, 0.005]

Abbreviations: CI, confidence interval; NDVI, Normalized Difference Vegetation Index; NO_2_, nitrogen dioxide; PM_2.5_, particulate matter of diameter ≤2.5 μm; ppb, parts per billion; SA1, Statistical Area 1; *β*, regression coefficient.

^**†**^ Percentage of population with stroke was transformed using square root (due to non‐normal distribution).

^‡^ Covariates: population density (persons/km^2^); decile of Index of Relative Social Advantage and Disadvantage, percentage of population aged ≥45 years who are female, percentage of population aged ≥65 years.

^§^ Population density and greenery were highly correlated.

* *P* < 0.05;

** *P* < 0.01;

*** *P* < 0.001 (in Bold).

### Interaction between walkability and PM_2.5_

Associations between walkability and all three cardiometabolic disease prevalences were highly significant in the univariable model, but not in the hypothesized direction (Table 2). However, when an interaction term for walkability and PM_2.5_ was included, the associations of PM_2.5_ and walkability with diabetes prevalence were statistically significant and in the hypothesized direction (Table 3), and the interaction term itself was significantly associated with diabetes prevalence (Table 3). There was no significant interaction of walkability and PM_2.5_ in relation to prevalence of heart disease or stroke; nor was there any significant interaction between walkability and NO_2_ in relation to any of the cardiometabolic disease prevalences (results not shown).

**Table 3:** Exploring interaction between walkability and particulate matter of diameter ≤2.5 μm in relation to the prevalence of diabetes

Exposure	Diabetes prevalence *β* [95% CI]
PM_2.5_ (μg/m^3^)	*β* = 0.87 [0.04, 1.69]*
Walkability (Walk Score)	*β* = −0.29 [−0.47, −0.11]**
Interaction PM_2.5_ × walkability	*β* = 0.03 [0.01, 0.05]**

*Note*: Linear regression model adjusted for covariates: population density (persons/km^2^), decile of Index of Relative Social Advantage and Disadvantage, percentage of population aged ≥45 years who are female, percentage of population aged ≥65 years.

Abbreviation: CI, confidence interval; PM_2.5_, particulate matter of diameter ≤2.5 μm; *β*, regression coefficient.

* *P* < 0.05;

** *P* < 0.01.

**Figure 1:**
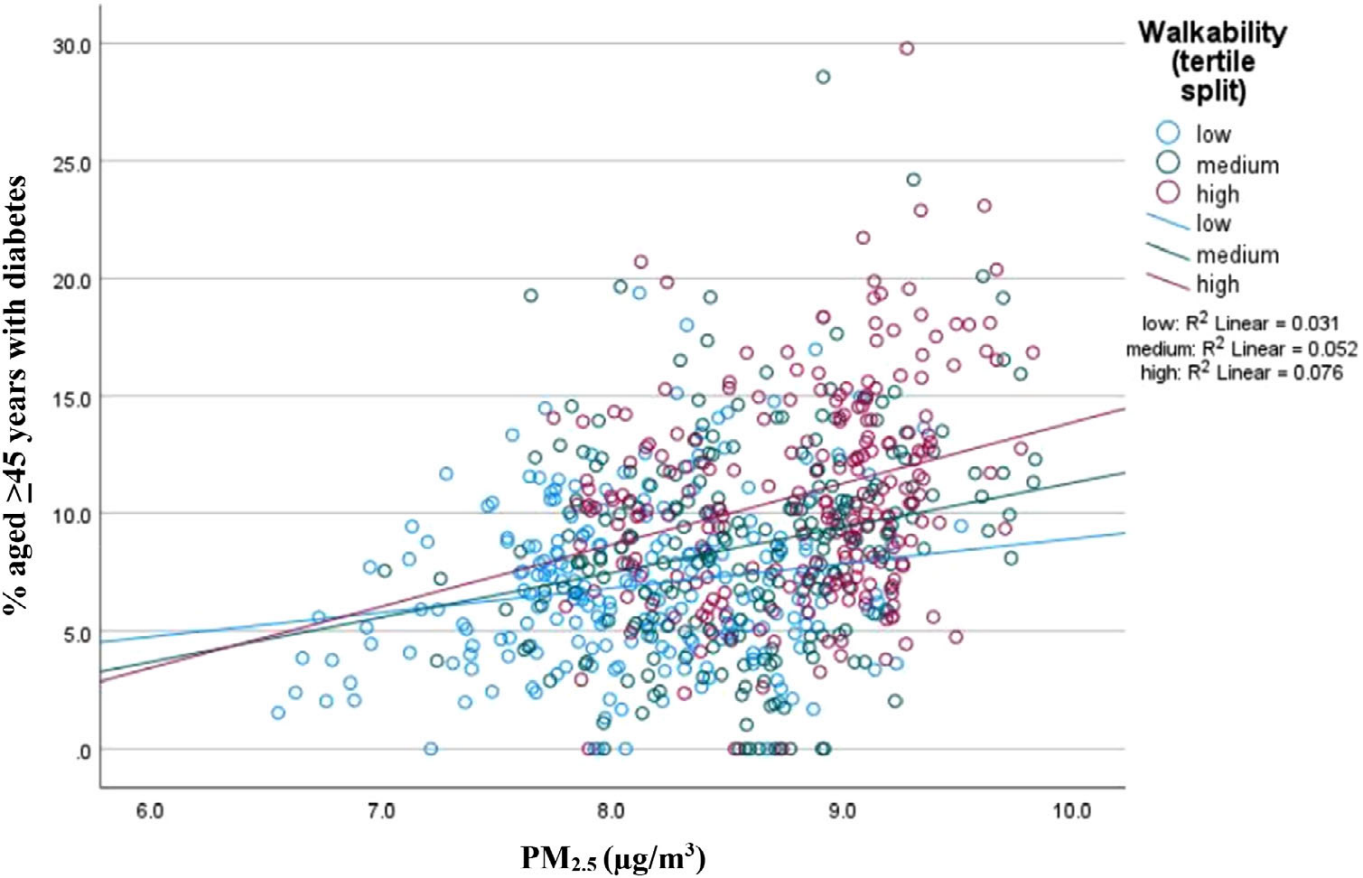
Interaction between walkability and fine particulate matter of diameter ≤2.5 μm (PM_2.5_) in relation to the prevalence of diabetes. This plot is a visual presentation of the interaction of walkability (measured using Walk Score) and PM_2.5_ (measured in μg/m^3^) in relation to the prevalence (%) of diabetes in neighborhoods. Walkability values have been categorized as “low”, “medium” or “high” based on a tertile split.

Walkability was re‐categorized as “low,” “medium” or “high” using a tertile split, and the interaction of walkability and PM_2.5_ in relation to prevalence of diabetes was plotted (Figure 1). This visualization of data shows that relatively higher walkability was associated with lower diabetes prevalence, only where PM_2.5_ levels were lower (Figure 1).

### Mediation analysis

Mediation analysis was carried out where greenery was significantly associated with the cardiometabolic disease prevalence, after controlling for covariates (therefore, not carried out for heart disease). Results of mediation analyses that explored PM_2.5_ and NO_2_ as potential mediators of associations between greenery and diabetes prevalence are presented in Table 4. A significant mediated effect was found in each case; the proportion mediated by PM_2.5_ was 92%, and the proportion mediated by NO_2_ was 69%. No mediated effects were found for PM_2.5_ and NO_2_ in the association between greenery and stroke prevalence (square root transformed), as pathway “b” (between the air pollutant and stroke prevalence, adjusting for greenery) was non‐significant (*P* ≥ 0.05; results not shown).

**Table 4:** Mediation analysis: adjusted^†^ associations of the a, b, c, and c^/^ pathways^‡^ and significant mediated effect by particulate matter of diameter ≤2.5 μm, and by nitrogen dioxide

Pathway c [NDVI➔ diabetes]	Pathway a [NDVI ➔ PM_2.5_]	Pathway b [PM_2.5_ ➔ diabetes adjusted for NDVI]	Pathway c^/^ [NDVI ➔ diabetes adjusted for PM_2.5_]	Mediated effect	Proportion mediated
Adjusted beta^**†**^ [95% CI]	Adjusted beta^**†**^ [95% CI]	Adjusted beta^**†**^ [95% CI]	Adjusted beta^**†**^ [95% CI]	a × b [95% CI]	(%)
−5.85 [−9.53, −2.17]**	−3.84 [−4.31, −3.38]***	1.40 [0.80, 2.00]***	−0.47 [−4.77, 3.84]	−5.38 [−7.84, −3.03]*	92%

Pathway c [NDVI➔ diabetes]	Pathway a [NDVI ➔NO_2_]	Pathway b [NO_2_➔ diabetes adjusted for NDVI]	Pathway c^/^ [NDVI ➔ diabetes adjusted for NO_2_]	Mediated effect	Proportion mediated
Adjusted beta^†^ [95% CI]	Adjusted beta^†^ [95% CI]	Adjusted beta^†^ [95% CI]	Adjusted beta^†^ [95% CI]	a × b [95% CI]	(%)
−5.85 [−9.53, −2.17]**	−10.36 [−11.60, −9.13]***	0.39 [0.16, 0.62]***	−1.78 [−6.24, 2.68]	−4.02 [−6.47, −1.65]	68.7%

Abbreviation: CI, confidence interval; IRSAD, index of relative socioeconomic advantage and disadvantage; NDVI, Normalized Difference Vegetation Index; NO_2_, nitrogen dioxide; PM_2.5_, particulate matter of diameter ≤2.5 μm.

^†^ Analyses were adjusted percentage of women among those aged ≥45 years, percentage of population aged ≥65 years and IRSAD decile.

^‡^ Pathways are shown in Appendix II, Figure A3.

* *P* < 0.05;

** *P* < 0.01;

*** *P* < 0.001.

## Discussion

The present study makes an important contribution to the growing body of research that examines neighborhood measures of greenery, PM_2.5_, NO_2_ and walkability in multi‐exposure models of cardiometabolic disease prevalence among mid‐to‐older aged adults. Our findings extend and further explore those from earlier studies that examined either greenery or air pollution or walkability in relation to cardiometabolic disease, in the absence of the other exposures.

The present findings that PM_2.5_ and NO_2_ were each positively associated with diabetes prevalence in single exposure models concurred with those of an Italian study.^38^ In our study, the positive association with diabetes prevalence remained for PM_2.5_ after controlling for NO_2_; however, the association between NO_2_ and diabetes prevalence was no longer significant after controlling for PM_2.5_. This supports the argument that NO_2_ is, in effect, an indicator of other co‐pollutants from vehicle emissions.^39^

The strengths of the present study are the exploration of interaction between walkability and PM_2.5_, and investigation of air pollution exposures as mediators of association between greenery and diabetes prevalence. Some previous studies that showed associations of greenery with lower diabetes prevalence,^40^ lower hospitalization rates for cardiovascular disease,^41^ as well as lower risk of incidence of cardiovascular disease,^42^ did not include air pollution or walkability exposures. Chandrabose *et al*.^21^ proposed that more walkable neighborhoods have destinations to which some people still drive, creating vehicle emissions that adversely affect health. We found that higher walkability was associated with lower diabetes prevalence only in areas with lower levels of PM_2.5_. Similarly, the CANHEART study,^43^ one of few studies examining multiple environmental exposures and cardiometabolic disease, reported that beneficial associations of higher walkability with diabetes were attenuated in more highly‐trafficked areas with higher NO_2_ levels.^43^

Our identification of both PM_2.5_ and NO_2_ as mediators of the association between greenery and diabetes prevalence aligns with findings of a study of greenery, air pollution and cardiometabolic disease in the Netherlands.^11^ Possibly, greenery might offset air pollution by plant‐based filtering and/or dispersal of air pollutants,^44^ or by occupying land that might otherwise be used for roads.^45^ Objective measurement would be required for confirmation. The present data are cross‐sectional, thus precluding any temporal or causal inference. Furthermore, there is potential in cross‐sectional studies for mediating effects to be overestimated.^46^ It is suggested that for greater understanding of mediators (and of possible reverse causality), future studies should be longitudinal, with measurement of exposure variables, potential mediators and outcome variables at three or more time‐points.^46^ Only area‐level data rather than individual‐level data are included in our analyses and, therefore, individual‐level associations cannot be reported due to the ecological design.^47^ Consequently, we were unable to adjust for many individual‐level factors and behaviors (e.g. smoking) that might affect cardiometabolic health outcomes.

We found no significant associations between either PM_2.5_ or NO_2_ and prevalence of heart disease. This was in contrast to the findings of a systematic review linking both long‐ and short‐term exposure to raised levels of PM_2.5_ with higher risks of cardiovascular disease and heart failure,^48^ and evidence of a causal association between short‐term exposure to NO_2_ and ischemic heart disease.^39^ Possibly, the term “heart disease” included in the Census was too vague, and led to underreporting of some conditions (e.g. arrythmia). Diabetes and stroke might have been reported more accurately if respondents required insulin or had experienced a defined medical event, such as a stroke.

The present study found that PM_2.5_ and NO_2_ were each adversely associated with stroke prevalence, although these associations were no longer significant when adjusting for each other. Overall, associations of air pollution and stroke are less studied than their associations with cardiovascular disease or diabetes. However, a systematic review^39^ reported that short‐term exposures to PM_2.5_ or NO_2_ were associated with stroke‐related hospital admissions and deaths from stroke. A more recent review^49^ provided strong evidence to support that short‐ and long‐term exposures to PM_2.5_ were causally related to incidence of ischemic stroke, but there was inadequate evidence to infer corresponding relationships for NO_2._ ^49^

Further strengths of the present study include the analysis of relatively granular exposure data (at SA1 level) for PM_2.5_, NO_2_, greenery and walkability in single and multi‐exposure models for almost 700 geographically diverse neighborhoods, with >150 000 residents aged ≥45 years. However, the conduct of our study in a single region (i.e. the Frankston–Mornington Peninsula region) of Australia might limit the generalizability of findings to other populations. Further study limitations include its cross‐sectional design, self‐report of professionally‐diagnosed disease outcome measures and non‐use of age standardized prevalence. Future studies should explore the use of more comprehensive clinical data that might be available across a geographic region. One efficient method would be the use of linked electronic health records^22^ to objectively measure the prevalence and incidence of hospitalization due to cardiometabolic disease.

In conclusion, the present study contributes to evolving evidence that neighborhood greenery might be protective against diabetes and stroke, but air pollution appears detrimental. Walkable neighborhoods might promote, health but only if air pollution is lower. Longitudinal studies, including clinically‐confirmed diagnoses, rather than self‐report, are required to confirm our findings, and inform public health policy to promote healthy aging.

## Disclosure statement

The authors declare no conflict of interest.

## Supporting information


**Appendix I.** This contains two figures with maps. Figure A1 depicts the study area, i.e. Frankston ‐ Mornington Peninsula area. Figure A2 depicts the Significant Urban Area included in Greater Melbourne.

**Appendix II.** This describes the methods used in our study for mediation analysis. MacKinnon's product of coefficients test^37^ was used to examine PM_2.5_ and NO_2,_ as potential mediators of associations between greenery and disease prevalence.

## Data Availability

Data were derived from public domain resources.
