# Supplementary material for: Exploring associations of greenery, air pollution and walkability with cardiometabolic health in people at midlife and beyond
Source: Geriatr Gerontol Int. 2023 Dec 19;24(Suppl 1):208–14. doi: 10.1111/ggi.14743 (PMC11503538; doi:10.1111/ggi.14743)
Supplement: Supplementary file 1 — Appendix I. This contains two figures with maps. Figure A1 depicts the study area, i.e. Frankston ‐ Mornington Peninsula area. Figure A2 depicts the Significant Urban Area included in Greater Melbourne. [file GGI-24-208-s002.docx]

**Appendix I**


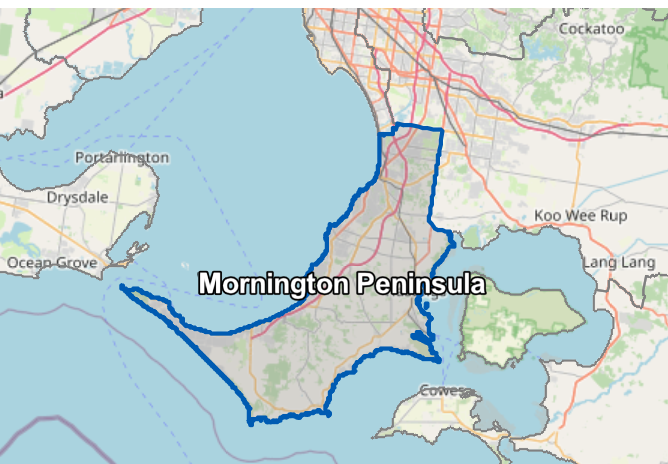


**Figure A1. Frankston - Mornington Peninsula area (Mornington Peninsula SA4)**

ABS Census 2021; Map data © OpenStreetMap contributors, CC-BY-SA


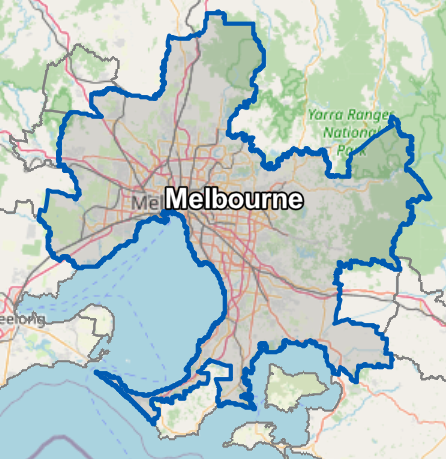


**Figure A2. Melbourne – Significant Urban Area**

ABS, Census 2021; Map data © OpenStreetMap contributors, CC-BY-SA
