# Supplementary material for: Exploring associations of greenery, air pollution and walkability with cardiometabolic health in people at midlife and beyond
Source: Geriatr Gerontol Int. 2023 Dec 19;24(Suppl 1):208–14. doi: 10.1111/ggi.14743 (PMC11503538; doi:10.1111/ggi.14743)
Supplement: Supplementary file 2 — Appendix II. This describes the methods used in our study for mediation analysis. MacKinnon's product of coefficients test 37 was used to examine PM2.5 and NO2, as potential mediators of associations between greenery and disease prevalence. [file GGI-24-208-s001.docx]

**Appendix II Mediation analysis**

MacKinnon’s product of coefficients test^1^ was used to examine PM_2.5_ and NO_2,_ as potential mediators of associations between greenery and disease prevalence (Figure A3). Pathway ‘c’ between greenery and disease prevalence is known as the total effect (Figure A3: I). These mediation analyses also examined associations between greenery and each TRAP variable (pathway ‘a’), and between each TRAP variable and disease prevalence adjusted for greenery (pathway ‘b’). The indirect or mediated effect (i.e., the product of ‘a’ and ‘b’) and corresponding 95% confidence interval were computed (Figure A3: II).^2^ This indicated how much of the association between greenery and the disease prevalence was attributable to TRAP. Some earlier methods of conducting mediation analysis required pathway ‘c’ (in addition to pathways ‘a’ and ‘b’) to be significant,^3, 4^ however, this is no longer mandatory.^1^ Conceptually, nonetheless, we were interested in examining mediation in cases where greenery was significantly associated with disease prevalence.


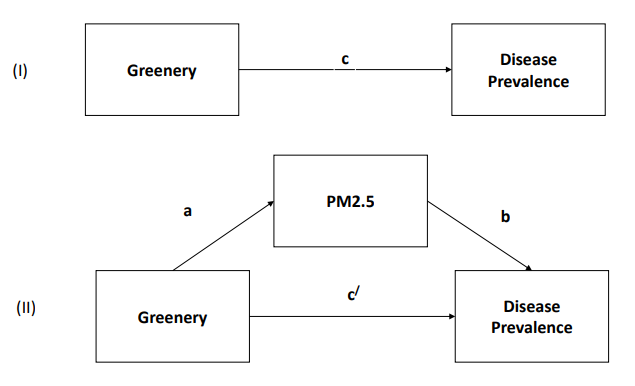


**Figure A3.** (I) The total effect of the independent variable (greenery) on the dependent variable (disease prevalence); (II) The indirect effect of the independent variable (greenery) on the dependent variable (disease prevalence) through the mediator variable (PM_2.5,_ a and b pathways in II) and the direct effect of the independent variable (greenery) on the dependent variable (disease prevalence), controlling for the mediator (PM_2.5,_ c^/^ pathway in II).

1. MacKinnon D. Introduction to statistical mediation analysis. Mahwah, NJ: Erlbaum; 2008.

2. Tofighi D, MacKinnon DP. RMediation: An R package for mediation analysis confidence intervals. Behavior Research Methods. 2011;43(3):692-700.

3. Baron RM, Kenny DA. The moderator-mediator variable distinction in social psychological research: Conceptual, strategic, and statistical considerations. Journal of Personality and Social Psychology. 1986;51(6):1173-82.

4. Fairchild AJ, McDaniel HL. Best (but oft-forgotten) practices: mediation analysis. The American journal of clinical nutrition. 2017;105(6):1259-71.
